# Unilateral pallidothalamic tractotomy at Forel's field H1 for cervical dystonia

**DOI:** 10.1002/acn3.51532

**Published:** 2022-03-08

**Authors:** Shiro Horisawa, Kotaro Kohara, Taku Nonaka, Atsushi Fukui, Tatsuki Mochizuki, Mutsumi Iijima, Takakazu Kawamata, Takaomi Taira

**Affiliations:** ^1^ Department of Neurosurgery Tokyo Women's Medical University Tokyo Japan; ^2^ Department of Neurology Tokyo Women's Medical University Tokyo Japan

## Abstract

**Background:**

Neurosurgical ablation of Forel's field H1 for cervical dystonia, which is currently abandoned, was formerly used in the 1960s–1970s. Regardless of the lack of neuroimaging modalities and objective evaluation scales, the reported efficacy was significant. Although recent studies have reappraised the ablation of the pallidothalamic tract at Forel's field H1 for Parkinson's disease, the efficacy for cervical dystonia has not been investigated well.

**Methods:**

Data of 35 patients with cervical dystonia who underwent unilateral pallidothalamic tractotomy at Forel's field H1 were retrospectively analyzed. The Toronto Western Spasmodic Torticollis Rating Scale (TWSTRS) scores, the neck score of the Burke–Fahn–Marsden Dystonia Rating Scale (BFMDRS), and adverse events were evaluated preoperatively and at the last available follow‐up period.

**Results:**

The mean clinical follow‐up period was 13.9 ± 6.5 months. The mean TWSTRS total scores were 34.3 ± 14.0 preoperatively and 18.4 ± 16.5 at the last available follow‐up period (46.4% improvement, *p* < 0.0001). The BFMDRS neck score also improved significantly from 6.2 ± 2.9 preoperatively to 2.8 ± 2.8 at the last available follow‐up period (55.0% improvement on the neck score, *p* < 0.0001). Reduced hand dexterity in seven patients, hypophonia in five patients, dysarthria in four patients, and executive dysfunction in one patient were confirmed as adverse events at the last available follow‐up evaluation. One patient had postoperative hemorrhage.

**Conclusion:**

The current study confirmed significant improvement in TWSTRS total scores and BFMDRS neck scores at the 13.9‐month follow‐up after unilateral pallidothalamic tractotomy. The pallidothalamic tract in Forel's field H1 is expected to be an alternative treatment target for cervical dystonia.

## Introduction

The pallidothalamic tract (PTT), including the ansa lenticularis and lenticular fasciculus, is composed of fiber bundle relay of the basal ganglia–thalamo–cortical circuit, which connects the globus pallidus internus (GPi) with thalamic subnuclei including the ventrolateral and ventroanterior nuclei, and centromedian–parafascicular complex nucleus.^1^ The ansa lenticularis courses anteromedially and ventrally around the posterior limb of the internal capsule (PLIC) and subsequently reaches the Forel's field H (anterior to the red nucleus, medial to the subthalamic nucleus (STN)).^2^ The lenticular fasciculus arches over the STN through Forel's field H2, below the zona incerta (ZI), and subsequently enters Forel's field H.^2^ Both fibers course in different ways and merge at Forel's field H, and they enter into the thalamic nuclei through Forel's field H1.^2^

Ablation of Forel's field is called campotomy or Forel H‐tomy, which was used to treat Parkinson's disease (PD), epilepsy, and cervical dystonia in the 1960s–1970s.^3^, ^4^, ^5^ However, the efficacy and safety of these studies are unclear due to the lack of neuroimaging modalities and objective evaluation scales. With an elaborate anatomical investigation, Jeanmonod's group renamed campotomy pallidothalamic tractotomy and reported its efficacy for PD using radiofrequency and focused ultrasound.^6^, ^7^ Although recent studies have reappraised the PTT ablation at Forel's field H1 for Parkinson's disease,^6^, ^7^, ^8^, ^9^, ^10^ the efficacy for cervical dystonia remains unclear. Our previous preliminary results of pallidothalamic tractotomy for dystonia were applied to patients with mixed etiologies and previous contralateral pallidotomy, which showed an 83.6% improvement in the Burke–Fahn–Marsden Dystonia Rating Scale (BFMDRS).^11^ This study aimed to retrospectively analyze unilateral pallidothalamic tractotomy for cervical dystonia without previous surgery.

## Methods

### Patient population

The data of 35 patients with cervical dystonia who underwent unilateral pallidothalamic tractotomy at the Tokyo Women's Medical University Hospital between January 2019 and January 2021 were retrospectively collected and analyzed. All patients were refractory to botulinum toxin injections. Deep brain stimulation (DBS) was rejected for the following reasons: refuse to have a mechanical device implanted and difficulty accessing hospitals that manage DBS from the remote area.

### Clinical evaluation

Toronto Western Spasmodic Dysphonia Rating Scale (TWSTRS) scores, BFMDRS scores, and adverse events were evaluated preoperatively and at the last available follow‐up period. The TWSTRS is a validated rating scale for cervical dystonia, consisting of three subscales including severity (0–35), disability (0–30), and pain (0–20), with higher scores indicating greater impairment. The TWSTRS total score ranges from 0 to 85, with the three subscales' sum. The BFMDRS has a 0–20 score (higher score indicating greater impairment) consisting of subscales for eyes (0–8), speech/swallowing (0–16), mouth (0–8), neck (0–8), trunk (0–16), right arm (0–16), left arm (0–16), right leg (0–16), and left leg (0–16). The subscales for the arms and legs were divided into contralateral and ipsilateral surgical sides. Head MRI scans were performed before and immediately after surgery, and 3 months postoperatively.

### Surgery

#### Surgical procedure

Stereotactic planning was performed using the Leksell SurgiPlan (Elekta, Stockholm, Sweden) and Brainlab Elements software (Brainlab, Munich, Germany). Under local anesthesia, a Leksell stereotactic frame (Elekta, Stockholm, Sweden) was fixed to the patient's skull. T1‐weighted axial and T2‐weighted axial, and coronal MRI (1‐mm slice) were used to determine the target and CT scans (1‐mm slice) for correcting MRI distortion were used. Our tentative target of PTT was intended to be located in Forel's field H1, where the ansa lenticularis and lenticular fasciculus merge, corresponding to the thalamic fasciculus on the Morel atlas.^12^ The mammillothalamic tract (MTT) as the medial boundary and STN as the inferior boundary are key structures to confirm the PTT location. Both structures were clearly visualized as low‐intensity areas on T2‐weighted MRI. We adjusted the supero‐inferior and mediolateral coordinates for PTT according to the MTT and STN location on T2‐weighted MRI. Additionally, 3‐mm and 1‐mm intervals were placed laterally from the MTT and superiorly from the STN, respectively. The shape of the thalamic fasciculus on the Morel atlas is elongated posterolaterally, requiring two stereotactic targets. The medial PTT target was set at 7–10 mm laterally and 1.0–3.5 mm inferior to the midpoint of the anterior commissure–posterior commissure (AC–PC) and at the midpoint of the AC–PC. The lateral PTT target was set at 10–13 mm laterally, 0–2.0 mm inferior, and 1 mm posterior to the midpoint of the AC–PC. The operation was performed under local anesthesia without microelectrode recording. We used a monopolar radiofrequency probe (1.0 mm diameter tip with an uninsulated length of 4.0 mm) and a Leksell Neuro Generator (Elekta) to confirm impedance monitoring and induce macrostimulation and coagulation. Coagulation was performed at 70°C for 40 sec for each target.

#### Surgical side

The contralateral side to the direction of head deviation was chosen for torticollis as the surgical side. For laterocollis, the contralateral side to the direction of neck tilting was chosen as the surgical side. For anterocollis and retrocollis, the contralateral hemisphere to the symptomatic dominant side, which was the more affected side of dystonia, including limb dystonia, was chosen as the surgical side.

### Lesion evaluation

Lesion evaluation was confirmed using the Brainlab Elements software (Brainlab, Munich, Germany). The distances from the center of the medial and lateral lesions created to the midline (mediolateral), midcommissural point (anteroposterior), and the AC–PC plane (dorsoventral) were measured by postoperative T1/T2‐weighted MRI on the day of surgery. Lesion volume (the total of medial and lateral lesions) was calculated using the low‐intensity area of the T2‐weighted MRI on the day of surgery.

### Statistical analysis

For non‐normally distributed data, the Wilcoxon signed‐rank test was used to compare the preoperative TWSTRS total and subscale scores, and the BFMDRS total and neck scores with those at the last available follow‐up. All statistical analyses were performed using SPSS (version 25.0; SPSS Inc., Chicago, IL, USA). All statistical tests were two‐tailed, and significance was set at *p* < 0.05.

### Ethical consideration

The ethics committee of the Tokyo Women's Medical University approved this study, and patient consent was waived owing to the study's observational nature.

## Results

### Patient demographics

Table 1 shows the patients' demographic characteristics. Altogether, 35 patients were included in this study (male, 22; female, 13). The mean age at the onset of dystonia and at surgery were 44.6 ± 10.7 and 54.0 ± 10.3 years, respectively. The mean clinical follow‐up period was 13.9 ± 6.5 months. Right‐ and left side surgeries were performed in 16 and 19 patients, respectively. The type of cervical dystonia and the surgical side is shown in Table 2.

**Table 1:** Patient characteristics.

Number of patients	35	
Male	22	
Female	13	
Age at onset	44.6 ± 10.7	(Range: 26–72)
Age at surgery	54 ± 10.3	(Range: 33–75)
Distribution of dystonia		
Cervical dystonia	21	
Segmental dystonia	10	
Generalized dystonia	4	
Movement type of cervical dystonia		
Tonic	25	
Phasic	10	
Side of surgery		
Right	16	
Left	19	
Follow‐up period	13.8 ± 6.6	(Range: 5–36)

**Table 2:** The type of cervical dystonia and the surgical side.

Type of cervical dystonia	The number of affected patients	Side of surgery
Rt torticollis	6	Left
Rt torticollis, Rt laterocollis	3	Left
Rt torticollis, Lt laterocollis	2	Left
Rt torticollis, Anterocollis	1	Left
Rt torticollis, Retrocollis	3	Left
Rt laterocollis	1	Left
Rt laterocollis, Retrocollis	1	Left
Lt torticollis	5	Right
Lt torticollis, Lt laterocollis	1	Right
Lt torticollis, Rt laterocollis	1	Right
Lt torticollis, Anterocollis	2	Right
Lt torticollis, Retrocollis	4	Right
Lt laterocollis	2	Right
Anterocollis, Rt arm dystonia	1	Left
Retrocollis, Lt arm dystonia	2	Right

### Efficacy

**Figure 1:**
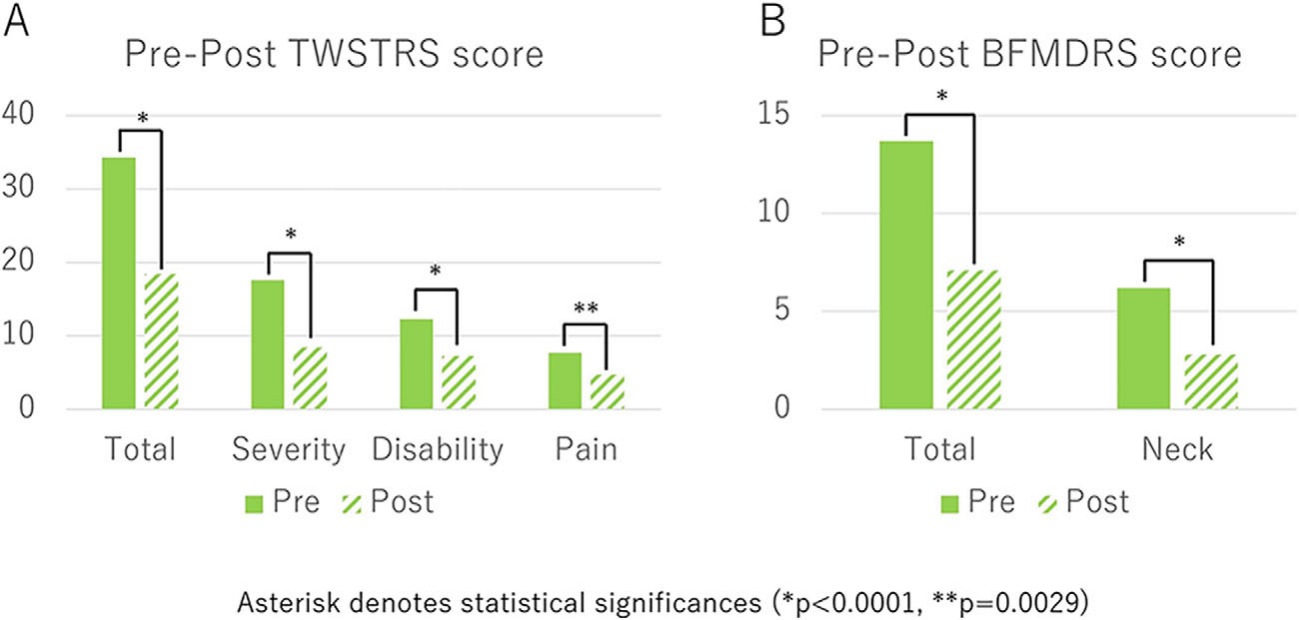
Pre‐ and postoperative TWSTRS and BFMDRS scores. Total, severity, disability, and pain scores of pre‐ and postoperative TWSTRS (A) and BFMDRS (B) were significantly improved after the surgery. Asterisks denote statistical significance (*p < 0.0001, **p = 0.0029).

**Figure 2:**
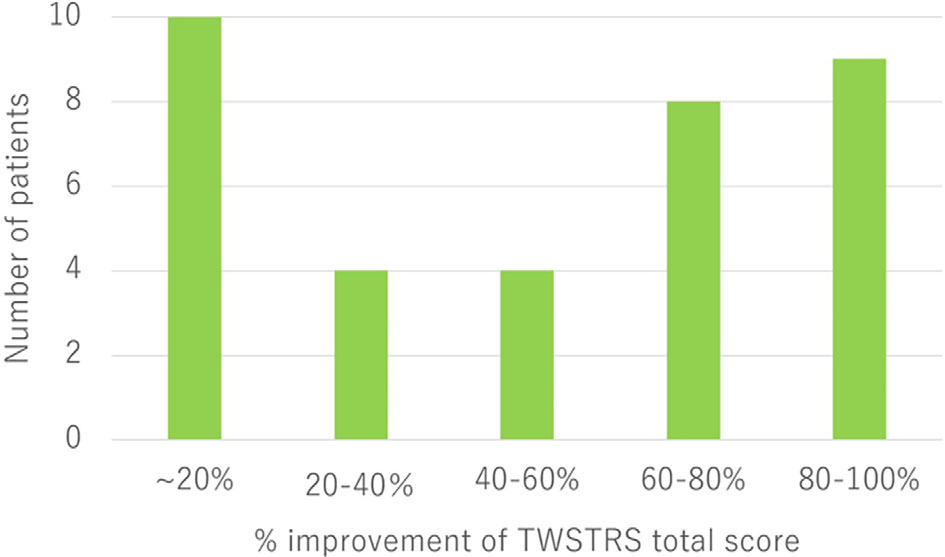
Distribution of improvement rate of TWSTRS total score. Improvement of TWSTRS total scores by 80%–100%, 60%–80%, 60%–40%, 40%–20%, and 20% was confirmed in nine patients (25.7%), eight patients (22.9%), four patients (11.4%), four patients (11.4%), and 10 patients (28.6%), respectively.

The TWSTRS total and three subscale scores significantly decreased from the preoperative to the last available follow‐up period. The mean TWSTRS total scores were 34.3 ± 14.0 preoperatively and 18.4 ± 16.5 (46.4% improvement, *p* < 0.0001) at the last available follow‐up period (Fig. 1A). The total score improved significantly from 13.7 ± 9.8 to 6.2 ± 2.9 preoperatively to 7.1 ± 7.6 and 2.8 ± 2.8 (50.5% improvement on the neck score, *p* < 0.0001, and 55.0% improvement on the neck score, *p* < 0.0001) at the last available follow‐up period (Fig. 1B). The detailed TWSTRS and BFMDRS subscale scores are shown in Table 3. The number of patients categorized by the degree of TWSTRS total score improvement at 20% intervals is shown in Figure 2. Nine patients (25.7%) showed 80%–100% improvement in TWSTRS total score, including five patients (14.2%) with 100% improvement. Ten patients (28.6%) showed <20% improvement in the TWSTRS total score (Fig. 2). Pre‐ and postoperative videos with excellent and poor results are shown in Videos S1 and S2, respectively.

**Table 3:** Clinical outcomes of unilateral pallidothalamic tractotomy.

	Number of affected patients	Pre	Post	% improvement	*p* value
TWSTRS					
Total	35	34.3 ± 14.0	18.4 ± 16.5	47.5%	<0.0001
Severity	35	17.6 ± 6.5	8.6 ± 7.5	51.1%	<0.0001
Disability	35	12.3 ± 7.1	7.3 ± 7.4	40.7%	<0.0001
Pain	21	7.7 ± 3.3	4.6 ± 5.0	40.3%	0.0029
BFMDRS					
Total	35	14.2 ± 9.9	7.0 ± 7.6	50.7%	0.0001
Neck	35	6.2 ± 2.8	2.8 ± 2.8	54.8%	0.0001
Eyes	11	5.8 ± 7.1	3.2 ± 3.9	44.8%	
Speech/Swallowing	8	3.5 ± 3.6	2.4 ± 2.4	31.4%	
Mouth	7	3.1 ± 2.3	1.7 ± 1.4	45.1%	
Trunk	13	5.5 ± 2.5	2.1 ± 2.4	61.8%	
Contralateral arm	11	6 ± 4.0	1.1 ± 2.1	81.7%	
Ipsilateral arm	7	4.1 ± 2.0	5.6 ± 4.6	−36.6%	
Contralateral leg	6	4.5 ± 6.0	0	100%	
Ipsilateral leg	4	3 ± 2.4	3 ± 2.4	0%	

### Adverse events

**Figure 3:**
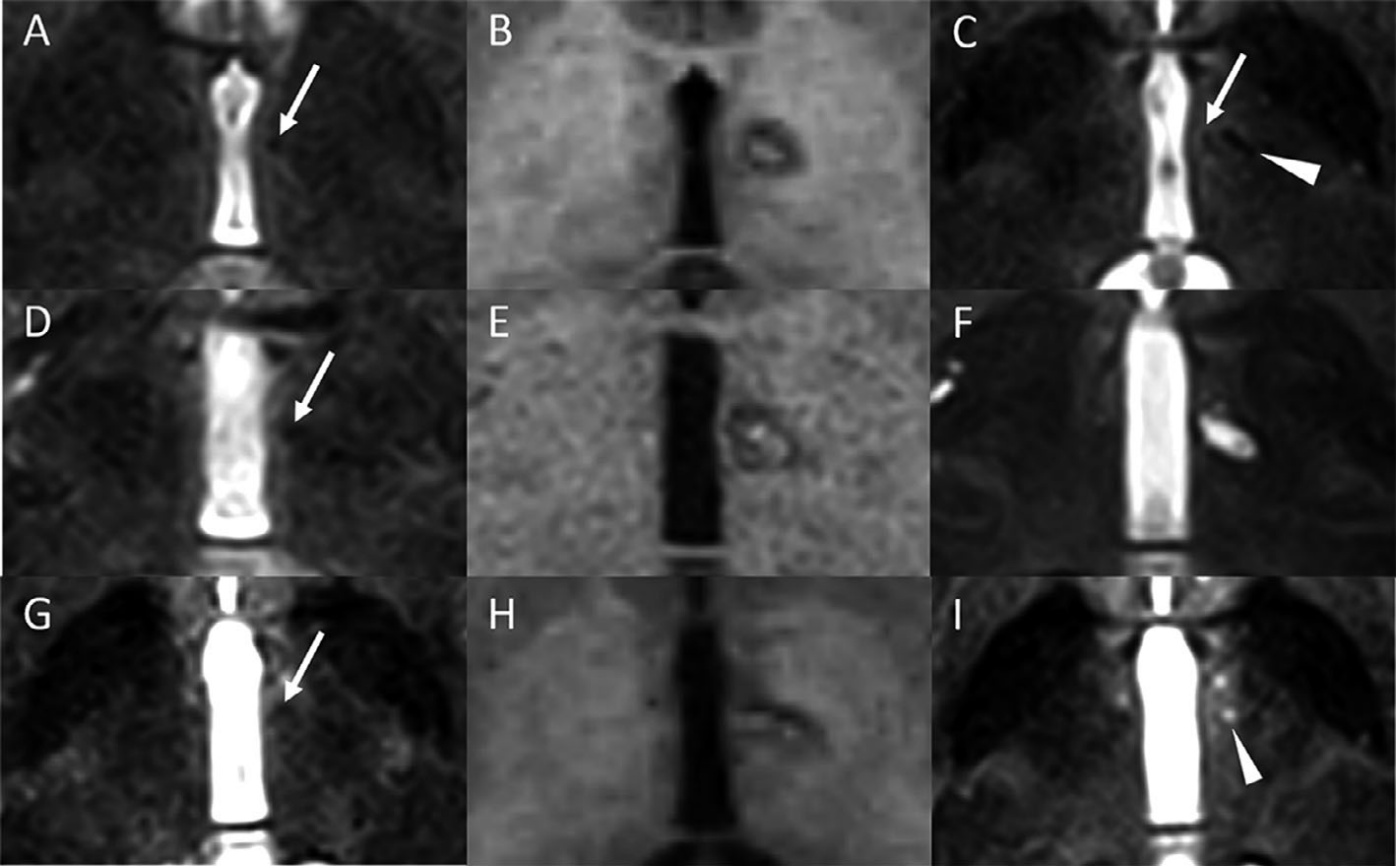
Pre‐ and postoperative MRI (day 0 and 3 months). (A–C) (successful case): Arrow showing left mammillothalamic tract (MTT) on the T2‐weighted MRI before the surgery. (A) Postoperative T1‐weighted MRI on the day of the surgery showing correct lesion location. (B) The 3‐month postoperative T2‐weighted MRI showing old lesion (arrowhead) and MTT (arrow). (C) D–F (hemorrhage case): Arrow showing left MTT on the T2‐weighted MRI before the surgery (D). Postoperative T1‐weighted MRI on the day of the surgery showing hemorrhage medially deviated. (E) The 3‐month postoperative T2‐MRI showing old scar involved MTT. (F) G–I (transient amnesia case): Arrow showing left MTT on the T2‐weighted MRI before the surgery. (G) Postoperative T1‐weighted MRI on the day of the surgery showing lesion medially deviated. (H) The 3‐month postoperative T2‐MRI showing old scar (arrowhead) and MTT is invisible (I).

The adverse events are shown in Table 4. One hemorrhagic complication (2.9%) extended medially, encroaching on the left side MTT. This patient suffered from prolonged executive dysfunction, such as recall disturbance of working procedures at 6 months postoperatively. Reduced hand dexterity in seven patients (20%), hypophonia in five patients (14.3%), dysarthria in four patients (11.4%), and executive dysfunction in one patient (2.9%) were confirmed as adverse events at the last available follow‐up evaluation. All the patients' reduced hand dexterity developed in the contralateral arm of the surgical side and were observed as micrographia, decreased writing speed, or difficulty in writing or typing. All hypophonia were mild and did not interfere with their daily lives. One out of four patients with dysarthria suffered from moderate dysarthria, and the remaining patients had very mild dysarthria. One patient experienced transient amnesia, which showed recall disturbance of recent events and spontaneously improved within 3 months postoperatively (Fig. 3E and F). Transient somnolence developed in two patients immediately after lesioning on the operating table. It completely resolved the next morning.

**Table 4:** Adverse events.

Dysarthria	4
Hypophonia	5
Reduced hand dexterity	7
Executive dysfunction	1
Transient amnesia	1
Transient somnolence	2

### Lesion location

A detailed analysis of the lesion and lesion volume is shown in Table 5. Representative preoperative T2‐weighted axial MRI images, postoperative T1‐weighted axial, and T2‐cor 0onal MRI images are shown in Figure 3A–C. Two patients had lesion encroachment in the MTT assay. One patient with hemorrhage encroachment on the left side MTT showed an old scar on the left side MTT on the 3‐month postoperative MRI (Fig. 3D–F). Another patient with transient amnesia showed lesion misalignment that encroached on the left side MTT on the immediate postoperative MRI (Fig. 3G–I). However, an old scar was almost invisible in the area of the left side MTT on the 3‐month postoperative MRI.

**Table 5:** Lesion evaluation.

Total lesion volume, mm^3^	47.1 ± 16.9
Lesion localization	
Mediolateral plane (distance from lesion to the midline), mm	
Medial target	8.9 ± 1.5
Lateral target	11.5 ± 1.2
Anteroposterior plane (distance from lesion to midcommissural point), mm	
Medial target	−0.3 ± 0.6
Lateral target	−1.0 ± 0.8
Dorsoventral plane (distance from lesion to anterior–posterior commissure plane), mm	
Medial target	−2.0 ± 0.8
Lateral target	−1.0 ± 1.0

## Discussion

Here, unilateral pallidothalamic tractotomy significantly improved overall cervical dystonia by 46.4%, severity by 51.4%, disability by 40.6%, and pain scores of the TWSTRS by 40.0% at the final follow‐up period (mean 13.8 ± 6.6 months). The total and neck scores of the BFMDRS also significantly decreased by 50.5% and 55.0%, respectively. Prolonged neurological deficits were found in five patients with hypophonia, seven patients with reduced hand dexterity, four patients with dysarthria, and one with executive dysfunction resulting from hemorrhagic complications.

Before the advent of the DBS era, there were several stereotactic lesioning targets for the treatment of cervical dystonia, including ZI, GPi, Forel's field H1, ventro‐oral internus (VOI), and the interstitial nucleus of Cajal (INC).^13^ Lesioning on those targets was mainly unilateral to avoid severe complications associated with bilateral lesioning. Despite the lack of MRI or CT images and objective evaluation scales such as the TWSTRS or BFMDRS, a certain number of patients responded well to unilateral lesioning surgery including Forel's field H1 with long‐term effects.^14^, ^15^ The rationale of unilateral ablation of Forel's field H1 by Hassler and Dieckmann for cervical dystonia is based on the concept that cervical dystonia resulted from imbalanced pallidal output due to unilateral disinhibited pallidum; unilateral pallidum is overactivated and turns the head to the contralateral side.^5^ To redress the imbalanced pallidal output, they considered it necessary to interrupt the efferent pathways of the disinhibited pallidum in the Forel's field H1 bundle. Recent neurophysiological and neuroimaging studies have shown interhemispheric differences in local field potential, cortical excitability, and intracortical inhibition confirmed in asymmetrical cervical dystonia, suggesting an imbalanced pallidal output. Lateralized local field potential oscillations, which reflect local neuronal activity in the GPi were observed in patients with torticollis and laterocollis, but not in those with retrocollis.^16^, ^17^ Interestingly, GPi discharge rates ipsilateral to the side of head turning were higher than contralateral and showed a positive correlation with torticollis symptom severity measured by TWSTRS.^17^ Abnormalities in cortical excitability and intracortical inhibition, which form the pathophysiological basis of dystonia. In horizontal rotational cervical dystonia, they are lateralized in the hemisphere contralateral to the direction of head deviation.^18^ Unilateral intracranial structural lesions including infarction, hemorrhage, and tumors can develop asymmetric cervical dystonia ipsilateral and contralateral to those lesions.^19^, ^20^, ^21^, ^22^ The surgical side that provides better results remains unknown. Functional neurosurgical predecessors have focused on the association between sternocleidomastoid muscle (SCM) and head rotation. The SCM is the large and superficial cervical muscle, whose primary function is head rotation to the opposite side. Hassler and Dieckmann have demonstrated that pallidothalamic stimulation increased the electromyographic activity in the ipsilateral SCM.^5^ Animal studies have reported that stimulation of the globus pallidus–entopeduncular nucleus (corresponding to the GPi in humans) increases neuronal firing rates of the ipsilateral SCM.^14^, ^23^ Clinical studies have reported that SCM weakness caused by ipsilateral hemispheric strokes and injection of barbiturates into the unilateral carotid artery.^24^, ^25^ Hassler and Dieckmann selected the contralateral side to the head rotation as the surgical side, which we followed.

In 1970, Hassler and Dieckmann used ablation of Forel's field H1 and VOI unilaterally in 21 patients with cervical dystonia.^14^ While 57% and 43% of the patients showed good and moderate outcomes, respectively, paresis was observed in three patients and ataxic and dyssynergia in 10 patients. In 1982, Hassler and Dieckmann et al. again reported unilateral lesioning using Forel's field H1, VOI, and ventro‐oral anterior nucleus in 87 patients with cervical dystonia.^23^ The outcomes were classified as excellent (completely abolished, 35%), good (32%), fair (23%), and unchanged (10%) with a 5.3‐year follow‐up. Interestingly, the distributions of their patient outcomes were similar to those in our study (Fig. 2). Postoperative persistent neurological deficits were motor neglect in 14 patients (16.1%), dyssynergia in two patients (2.3%), and hemiparesis in two patients (2.3%). They described that motor neglect was confirmed in the extremities contralateral to the side of the lesion, which reduced spontaneous activity and an inability to perform fine movements. Motor neglect and dyssynergia corresponded to reduced hand dexterity in the present study. In 1972, Mundinger et al. reported the efficacy of unilateral lesioning using several targets, including VOI (23 patients), ZI including Forel's field H1 (19 patients), GPi (seven patients), INC (one patient), and VOI/ventral intermediate/ventral caudal nuclei (one patient) with up to a 6‐year follow‐up period.^15^ Patients treated with ablation of ZI, including Forel's field H1, achieved the best symptomatic improvement (61% good), followed by VOI (36% good) and GPi (50% fair). Anatomical ablation of ZI inevitably includes neighboring structures including Forel's field H1 and H2 which implies that Forel's field H1 or H2 might be responsible for the beneficial effect on cervical dystonia in this study. Loher and Krauss have reported on Mundinger's unpublished larger series of stereotactic lesioning surgery for cervical dystonia,^13^ wherein 92 and 11 patients underwent unilateral and bilateral lesioning, respectively. The targets included Zi (27%) and Zi with Vo nucleus (56%). Good outcomes were described in 63% of the patients who underwent Zi lesioning and 48% of those who underwent Zi and Vo lesioning. Adverse effects were described in 68% of patients undergoing bilateral procedures and 40% of patients with unilateral procedures. Limb paresis and dysarthria developed in 16% and 15% of patients, respectively. Moll et al. have reported an interesting case of cervical dystonia who underwent unilateral subthalamotomy using Mundinger's approach 30 years ago, which provided complete resolution of cervical dystonia.^26^ For 30 years postoperatively, the patient was free from cervical dystonia. The head MRI 32 years postoperatively revealed that the lesion mainly focused on Forel's field H and the posterior subthalamic area. They stated that the interruption of the PTTs at the level of the ZI was the most prominent anatomical structure that might be responsible for the beneficial effect in this case.

Pallidotomy or pallidal DBS targets the posteroventrolateral part of GPi which is strongly associated with the origins of pallidothalamic fibers.^1^ Regarding neuromodulation of pallidal output to the thalamus, pallidotomy, and pallidothalamic tractotomy may provide a similar symptomatic improvement on dystonia. However, the possible adverse effects of pallidotomy or pallidothalamic tractotomy are different because of the surrounding structures. In 69 patients with dystonia who underwent unilateral pallidotomy, delayed cerebral infarction at the PLIC in three patients (4.4%), hemiparesis in two patients (2.9%), and visual disturbance in one patient (1.4%) were reported.^27^ These complications were strongly associated with injury to adjacent structures of the GPi, including the PLIC (medial to GPi) leading to hemiparesis and optic tract (caudal to GPi) leading to visual field disturbance. Parkinsonism, including bradykinesia, gait disorder, micrographia, and other parkinsonian motor disturbances, excluding tremor and rigidity, have been reported as stimulating adverse events in GPi‐DBS. The detailed mechanism of parkinsonism associated with GPi‐DBS and pallidotomy remains unknown. Stroke on the globus pallidus externus, laterally located at the GPi, can also induce parkinsonism.^28^ Delayed cerebral infarction at the PLIC, which may be associated with the involvement of perforating arteries from the middle cerebral artery, was also reported in 6% in another study, leading to serious permanent neurological deficits.^29^ PLIC is medially located to the GPi, and unexpected encroachment of the lesion or hemorrhage can also induce hemiparesis. In contrast, the PTT in Forel's field H1 does not have surrounding structures that can induce hemiparesis or visual field disturbance. However, careful attention should be paid to the MTT, in which injury can induce amnesia and the STN, inducing dyskinetic movements. Here, one patient showed prolonged executive dysfunction; another patient showed transient retrograde amnesia, both of which indicated that the medial border of the lesion unexpectedly encroached on the left side of the MTT. Moreover, it should be noted that decreased hand dexterity developed relatively often (20%) in this study. All seven patients noticed reduced hand dexterity in their dominant hand contralateral to the surgical side when writing, typing, or performing fine manual tasks. The present retrospective study may have a high potential for overlooking reduced hand dexterity in the nondominant hand contralateral to the surgical side. Compared with DBS, ablative procedures are likely to induce more adverse events often. In a comparative study of tremor surgery, including DBS and radiofrequency ablation, ataxia/gait, paresthesia, and hemiparesis developed more commonly in the radiofrequency ablation group than in the DBS group.^30^ However, complications after lesioning procedure are likely to improve over time. In a retrospective analysis of focal hand dystonia managed with unilateral ventro‐oral thalamotomy, dysarthria, and weakness developed in 12 and 11 patients, respectively.^31^ During the 25.4‐month follow‐up, the condition of 10 (83.3%) and 9 (81.8%) patients improved, respectively.^31^ Longer follow‐up studies are required to evaluate prolonged neurological deficits associated with the ablative procedure.

Recent studies of pallidothalamic tractotomy have been reported only from three centers, including Switzerland, Brazil, and Japan.^6^, ^8^, ^11^ Our lesion location was determined by reference to the PTT location by Jeanmonod's group. The target locations of the three centers were quite similar at 8 mm lateral from the third ventricle wall, 0–1 mm posterior to the midcommissural point, and 0–2.5 mm inferior to the midcommissural point. According to Morel's atlas, the width of the PTT (thalamic fasciculus) is 6 mm at the AC–PC plane.^12^ Therefore, we placed one lesion medially and another lesion laterally. Reported adverse events associated with pallidothalamic tractotomy were dysarthria, dysphonia, and transient psychiatric deterioration. Gallay et al. have reported hypophonia (six patients, 11.8%) and transient anxio‐depressive status (one patient, 2%) in 51 patients with PD treated by focused ultrasound pallidothalamic tractotomy.^6^ Godinho et al. have reported foot dystonia (one patient), transient apathy (two patients, 16.7%), transient hypersexuality (two patients, 16.7%), and transient somnolence (four patients, 33.3%) in 12 patients with PD treated by radiofrequency pallidothalamic tractotomy (authors called campotomy).^8^ Our previous study has shown prolonged neurological deficits, including hypophonia (two patients, 20%), decreased response to l‐dopa (three patients, 30%), fatigue (one patient, 10%), and transient somnolence (6patients, 60%) in 10 patients with PD after unilateral pallidothalamic tractotomy.^10^ Combined unilateral pallidotomy and contralateral palliodothalamic tractotomy‐induced hypophonia (six patients) and dysarthria (one patient) in 11 patients with dystonia.^11^ All reported adverse events associated with psychiatric deterioration were transient.^6^, ^7^, ^8^ Foot dystonia gradually faded over 30 days, in which lesion location was more lateral and inferior than intended.^8^ Thus, the involvement of STN may be the cause of foot dystonia. Interestingly, transient somnolence was only reported in studies of PD.^3^, ^6^, ^8^, ^10^ Spiegel et al. considered transiently decreased consciousness after pallidothalamic tractotomy (author called campotomy) resulting from lesion encroachment on the ascending reticular activating system. Other previous and current studies of pallidothalamic tractotomy for dystonia, Huntington's disease, and epilepsy did not report transient somnolence.^11^, ^32^, ^33^, ^34^, ^35^ Here, only two out of 35 patients had transient somnolence (5.7%).

The most frequently available stereotactic surgical target for cervical dystonia is GPi. A recent pooled meta‐analysis study has revealed that bilateral GPi‐DBS improved cervical dystonia by 60.4%, 54.8%, 67.7%, and 55.9% reduction of TWSTRS total, severity, disability, and pain score, respectively.^36^ We have previously reported that unilateral pallidotomy significantly improved cervical dystonia with a 47.9% reduction in TWSTRS total score at the 6‐month postoperative evaluation.^37^ Additionally, a recent study has shown that bilateral and unilateral pallidotomies improved cervical dystonia with 73% and 50% reduction on the neck subscale of BFMDRS, respectively.^27^ The STN is also an available target of DBS for cervical dystonia in a limited number of studies. The reported efficacy varies from 22.8% to 63.9% improvement in TWSTRS total score.^38^, ^39^, ^40^, ^41^ Compared to previous studies of stereotactic neurosurgical treatment using GPi or STN, the PTT at Forel's field H1 can be an alternative stereotactic surgical target for cervical dystonia. The efficacy of unilateral pallidothalamic tractotomy in the present study (46.3% improvement of TWSTRS total score) is similar to that of unilateral pallidotomy in our previous study (47.9% improvement of the TWSTRS total score).^37^ However, recurrence or deterioration of cervical dystonia may develop later after unilateral ablative procedures. Bilateral ablative procedures whose clinical benefits for cervical dystonia are better than those of unilateral ablative procedures are likely to induce irreversible complications such as dysarthria, dysphagia, and dysphonia. Additionally, our previous study showed serious postural instability and gait disturbances in two out of 20 patients who received bilateral pallidotomy. In the PD study, bilateral pallidothalamic tractotomy‐induced speech impairment, including hypophonia and dysarthria, in 50% of patients.^9^ In the present study, hypophonia, and dysarthria were relatively often even after unilateral pallidothalamic tractotomy. Bilateral pallidothalamic tractotomy is highly likely to induce irreversible serious speech impairment considering this available evidence. Bilateral DBS using GPi or STN is a balanced procedure that does not induce irreversible complications to ensure the compatibility between safety and efficacy. The target that is best for treating cervical dystonia remains unknown. Table 6 summarizes the reported outcomes and adverse events of GPi, STN, and PTT using ablation and stimulation. DBS of pallidothalamic tract at Forel's field H1 for dystonia was recently reported, but detailed clinical outcomes of cervical dystonia were not reported.^42^ Ablation of the isolated subthalamic nucleus for cervical dystonia has not been reported. Magnetic resonance‐guided focused ultrasound (MRgFUS), which makes focal lesioning without skin incision, is also available for pallidothalamic tract ablation.^6^, ^9^ However, possible complications associated with target ablation by MRgFUS are considered the same as those of radiofrequency ablation.^6^, ^8^, ^10^ MRgFUS will be useful to establish robust evidence with sham‐controlled trial, which is ethically impossible for radiofrequency ablation.

**Table 6:** Summary of DBS and ablation for cervical dystonia.

	DBS			Ablation		
	GPi^36^, ^43^, ^44^, ^45^	STN^39^, ^40^, ^41^, ^46^	PTT	GPi^27^, ^37^	STN	PTT (Present study)
Efficacy						
TWSTRS	60.4% (Bilateral)^1^	22.8–80.3% (Bilateral)	Not reported	47.9% (Unilateral)	Not reported	46.4% (Unilateral)
BFMDRS				50% (Unilateral) 73.1% (Bilatreal)		54.8% (Unilateral)
Target‐specific adverse events						
	Bradykinesia, Gait disturbance, Postural instability	Dyskinesia, Weight gain, Depression		Delayed cerebral infarction, Bradykinesia, Postural instability, Gait disturbance		Amnesia, Executive dysfunction
Common adverse events						
	Dysarthria, Dysphonia, Dysphagia					

DBS, deep brain stimulation; GPi, globus pallidus internus; STN, subthalamic nucleus; PTT, pallidothalamic tract; TWSTRS, Toronto Western Spasmodic Torticollis Rating Scale; BFMDRS, Burke–Fahn–Marsden Dystonia Rating Scale.

^1^ Clinical outcomes reported by pooled meta‐analysis.^36^

This study had several limitations. In the retrospective analysis, subtle adverse events were likely overlooked. One of the major concerns associated with lesioning surgery is the recurrence or temporary benefits. Because of the relatively short‐term follow‐up, long‐term efficacy could not be concluded in this study. Lastly, due to the lack of evaluations of cognitive and mood states, the safety profile of psychiatric or behavioral function is unknown.

## Conclusion

This study confirmed significant improvement in TWSTRS (46.4% reduction) and neck subscore of BFMDRS (55.0% reduction) at the 13.8‐month follow‐up after unilateral pallidothalamic tractotomy at Forel's field H1. The PTT in Forel's field H1 may be an alternative lesioning target for cervical dystonia.

## Conflict of Interest

The authors report no conflict of interest relative to the research covered in this manuscript.

## Author Contributions

SH: study design, patient selection, analysis of imaging data, interpretation and acquisition of data, writing, and revising the manuscript; KK: acquisition of data; TN: acquisition of data; AF: statistical analysis; TM: acquisition of data; MI: interpretation and acquisition of data; TK: study design; TT: study design, patient selection, acquisition, and interpretation of data.

## Ethics Approval

The Institutional Review Board at the Tokyo Women's Medical University approved this study (No.3576).

## Supporting information


**Video S1.** Pre‐post operative condition with excellent result.

**Video S2.** Pre‐post operative condition with poor result.

## Data Availability

Processed data and codes used in this study are available upon request from qualified investigators.
